# Longitudinal monitoring of load-velocity variables in preferred-stroke and front-crawl with national and international swimmers

**DOI:** 10.3389/fspor.2025.1585319

**Published:** 2025-05-07

**Authors:** Ryan Keating, Rodney Kennedy, Carla McCabe

**Affiliations:** School of Sport and Exercise Science, Ulster University, Belfast, United Kingdom

**Keywords:** swimming, male, female, semi-tethered, profiling, mixed-effect model

## Abstract

Load-velocity (LV) profiling in swimming provides key metrics, including theoretical maximal velocity (*V*_0_) and theoretical maximal load (*L*_0_); however, longitudinal studies tracking these variables across competitive seasons are limited. This study investigated LV profiling and competition performance in national and international-level swimmers (Level 1–3) over a 15-month period. Twenty-six swimmers participated (16 males: age: 19.8 ± 3.9 years, body mass: 80.3 ± 7.9 kg, height: 1.84 ± 0.07 m; 10 females: age: 20.7 ± 3.6 years, body mass: 68.2 ± 5.7 kg, height: 1.74 ± 0.03 m), all specializing in 50–200 m events. Swimmers completed 4–6 testing sessions, each involving 3 × 10 m sprints against resistances of 1, 5, and 9 kg (males) and 1, 3, and 5 kg (females), in both front-crawl and their preferred-stroke. Linear mixed-effects models assessed changes in LV outputs—*V*_0_, *L*_0_ (absolute and relative to body mass), relative slope (−*V*_0_/*rL*_0_), and active drag (AD). Smallest worthwhile change (SWC) assessed within-athlete variation, while Pearson's correlations evaluated relationships between race performance and LV outputs. Analysis of preferred-stroke found males exhibited significantly higher values across all variables except the slope (*p* = 0.607). National-level swimmers had lower *L*_0_ (−2.8 kg, *p* = 0.019), but no statistical difference in *rL*_0_ (−1.5%, *p* = 0.244) or slope (−0.002 m/s/%, *p* = 0.558). AD remained stable across observations, though males produced greater drag (+30.2 N, *p* < 0.001), while national-level swimmers produced less (−12.8 N, *p* = 0.045). Analysis of front-crawl performance found males presented higher values across all variables (*p* ≤ 0.05) while national-level swimmers were lower (*p* < 0.005). SWC analysis revealed that most within-athlete changes in *V*_0_ and *L*_0_ were trivial or unclear, with only isolated meaningful changes observed. Large to very large correlations existed between race performance and *L*_0_ (*r* = 0.67, *p* < 0.05), *V*_0_ (*r* = 0.73, *p* < 0.05), and AD (*r* = 0.58–0.7, *p* < 0.05) at select observations. These findings highlight the stability of LV profiling metrics over time while reinforcing their relevance in distinguishing between performance levels. This suggests their potential utility in talent identification and informing training prescription.

## Introduction

Swimming is a highly competitive sport that requires a complex interplay of physiological, biomechanical, and psychological factors to optimize performance (1, 2). The competitive swimmer's annual calendar typically consists of short-course (25 m) swimming from September to December followed by long-course (50 m) swimming from January to August, culminating in major international and national championships. To ensure peak performance at these key competitions, coaches meticulously periodize their training plans, making the identification of relevant performance-monitoring variables essential for optimizing performance.

Biomechanical assessment plays a crucial role in understanding swimming performance. Deterministic modelling suggests that free-swimming velocity is influenced by the ratio of stroke length and stroke rate, with improvements in stroke length largely dependent on maximizing propulsive forces while minimizing resistive forces (3). Fully-tethered swimming is a well-established method for assessing a swimmer's resultant propulsive force (4–6). In relation to sprint performance, peak and average forces obtained in front-crawl, backstroke, breaststroke and butterfly have shown strong correlations with swimming velocity in 50 m (*r* = −0.90 to −0.69 and −0.94 to −0.81, respectively), and 100 m (*r* = −0.89 to −0.73 and −0.93 to −0.78, respectively) (6–8). Additionally, impulse measured during front-crawl swimming has been positively correlated with 100 m time-trial performance (*r* = 0.76) (9). However, longitudinal data on fully-tethered swimming performance is scarce. One case study of an elite sprint swimmer reported improvements of 17, 12% and 31% in tethered swimming peak force, average force and impulse respectively, over three years (10), but did not investigate seasonal changes or their impact on competition performance, highlighting a key gap in the current literature.

An emerging alternative to fully-tethered swimming is semi-tethered swimming with load-velocity (LV) profiling, which offers a more context-specific assessment of a swimmer's in-water performance. LV profiling provides coaches and practitioners with key metrics such as maximal theoretical load (*L*_0_), which reflects the swimmer's ability to generate propulsive force, and theoretical velocity (*V*_0_), representing clean swimming speed (11). Advances in commercially available resistance devices have improved the reliability (12) and replicability of these assessments, enabling investigations across different level swimmers and strokes (11, 13–18). Additionally, LV profiling has been utilized to assess active drag (19, 20) and differentiate upper- and lower-body contributions to front-crawl swimming (21). However, most LV studies have been cross-sectional, with the longest investigating the effects of a 5-week cessation period on LV performance (22). Furthermore, most research has focused on front-crawl swimming, with limited investigation into preferred-stroke swimming. While front-crawl dominates training, even among swimmers specializing in other strokes, it remains unclear how these adaptations affect performance in their preferred event (23). To date, no longitudinal study has systematically examined seasonal variations in LV outputs across different strokes and their relationship with elite competition performance.

Despite the importance of tracking performance variables, longitudinal studies involving elite swimmers remain limited, with most existing research focusing on age-group athletes (24–28). A five-year study of 40 elite swimmers monitored changes in anthropometrics, physiology, and swim performance in a 7 × 200 m incremental step test (29). Variables assessed included body composition, stroke rate, stroke length, blood lactate, and heart rate. While this study offered valuable insight into multi-year training adaptations, it did not examine how these variables changed around competitions or their relationship with competition performance, leaving a gap in understanding how these factors influence peak performance. A subsequent investigation using the same testing protocol across a single competition season found no significant changes in velocity, stroke index, stroke length, or stroke frequency at 4 mmol of blood lactate across three time points (30). These findings suggest two possibilities: (i) commonly monitored physiological and biomechanical training variables may not be sufficiently sensitive to detect meaningful performance fluctuations within a competitive season, or (ii) at the elite level, these variables may have stabilized due to years of training, with further improvements relying on more technical, tactical, or psychological factors. This highlights the need to explore alternative or complementary performance metrics. Additionally, Costa et al.'s study was limited to male, 200 m front-crawl specialists, raising questions about the generalizability of these findings to female swimmers and other strokes and distances.

Therefore, this study aims to monitor longitudinal changes in LV outputs during both preferred-stroke and front-crawl swimming and assess their relationship with competition performance in national- and international-level swimmers. While preferred-stroke performance may influence competition outcomes, the high training volume of front-crawl may also impact LV outputs, warranting the assessment of both strokes. By comparing swimmers across performance levels, this study seeks to uncover how LV metrics relate to competitive success and to provide novel insights into seasonal biomechanical changes in elite swimming.

## Materials and methods

### Participants

Twenty-six swimmers from three National Performance Centers and one club participated in the study, including 16 males (age: 19.8 ± 3.9 years, body mass: 80.3 ± 7.9 kg, height: 1.84 ± 0.07 m, arm span: 1.92 ± 0.08 m) and 10 females (age: 20.7 ± 3.6 years, body mass: 68.2 ± 5.7 kg, height: 1.74 ± 0.03 m, arm span: 1.79 ± 0.05 m), specializing in 50–200 m events. All participants provided written consent after receiving details about the purpose, procedures, and associated risks. Their anthropometric data and performance levels are shown in Table 1. Using the recently proposed performance classification model (31), swimmers at Levels 1 and 2 were classified as international (*n* = 13), while those at Level 3 were classified as national (*n* = 13). Among the male participants, preferred strokes included front-crawl (*n* = 9), backstroke (*n* = 1), breaststroke (*n* = 3), and butterfly (*n* = 3). Among the female participants, preferred strokes were front-crawl (*n* = 3), backstroke (*n* = 4), and breaststroke (*n* = 3). The study was ethically approved by Ulster University.

**Table 1:** Age, anthropometrics and performance level of participants (mean ± SD).

	National		International*	
	Males (*n* = 8)	Females (*n* = 5)	Males (*n* = 8)	Females (*n* = 5)
Age (years)	17.6 ± 0.9	20.2 ± 4.9	22.0 ± 4.5	21.1 ± 2.1
Body mass (kg)	76.1 ± 6.9	64.9 ± 5.8	84.5 ± 6.7	71.5 ± 3.7
Height (m)	1.78 ± 0.05	1.72 ± 0.02	1.90 ± 0.04	1.77 ± 0.03
Arm span (m)	1.86 ± 0.06	1.76 ± 0.02	1.97 ± 0.04	1.82 ± 0.05
100 m FC SC world aquatic points	651 ± 93	676 ± 93	768 ± 69	756 ± 67
SC level	3	3	3	3
Preferred-stroke LC world aquatic points	744 ± 31	732 ± 47	861 ± 36	855 ± 37
LC level	3	3	2	2

FC, front-crawl; LC, long course competition; SC, short course competition.

* Students *T*-test found *p* < 0.05 for age, body mass, height and arm span.

### Design

**Figure 1:**
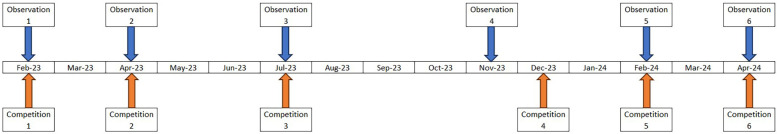
Timeline of observations and competitions over the study duration.

This study was a longitudinal, observational investigation. Each swimmer participated in four to six profiling sessions from February 2023 to April 2024. Profiling was scheduled within two weeks of identified competitions, which were international, national, or regional, depending on the athlete's goals and performance level. Most competitions were long-course, with short-course events occurring in December 2023. The competition and profiling schedule is outlined in Figure 1.

### Procedures

The procedure for LV profiling has been described in a previous study (Keating et al.). Participants performed their individual competition warm-up on land and in-water. At the start of each session, anthropometric measurements were recorded, including standing height (stadiometer, SECA 242, Hamburg, Germany), body mass (weighing scales, SECA 813, Hamburg, Germany), and arm span (measured from middle fingertip to middle fingertip across outstretched arms using Anthrotape, Rosscraft, Canada). Following 20 min of passive recovery (32), during which swimmers changed into their competition suit, they performed a series of 10 m maximal swims following a 5 m scull. Swimmers wore a swim belt (S11875BLTa, NZ Manufacturing, OH, USA) attached to a portable resistance device (1080 Sprint, 1080 Motion AV, Lidingö, Sweden) mounted on a start block. The height of the device was recorded each session for horizontal correction as part of data processing (11). The device was set to isotonic resistance, and eccentric and concentric velocity of 0.05 and 14 m/s, respectively. Females performed swims with 1, 3, and 5 kg of resistance, while males used 1, 5, and 9 kg. Four minutes of passive rest was given between trials. All participants completed front-crawl trials first, then repeated the process if their preferred-stroke was backstroke, breaststroke or butterfly.

### Data processing

#### Competition data

Times were converted to World Aquatic points to standardize performance across long course and short course competitions (33).

#### Load-velocity profiling

**Figure 2:**
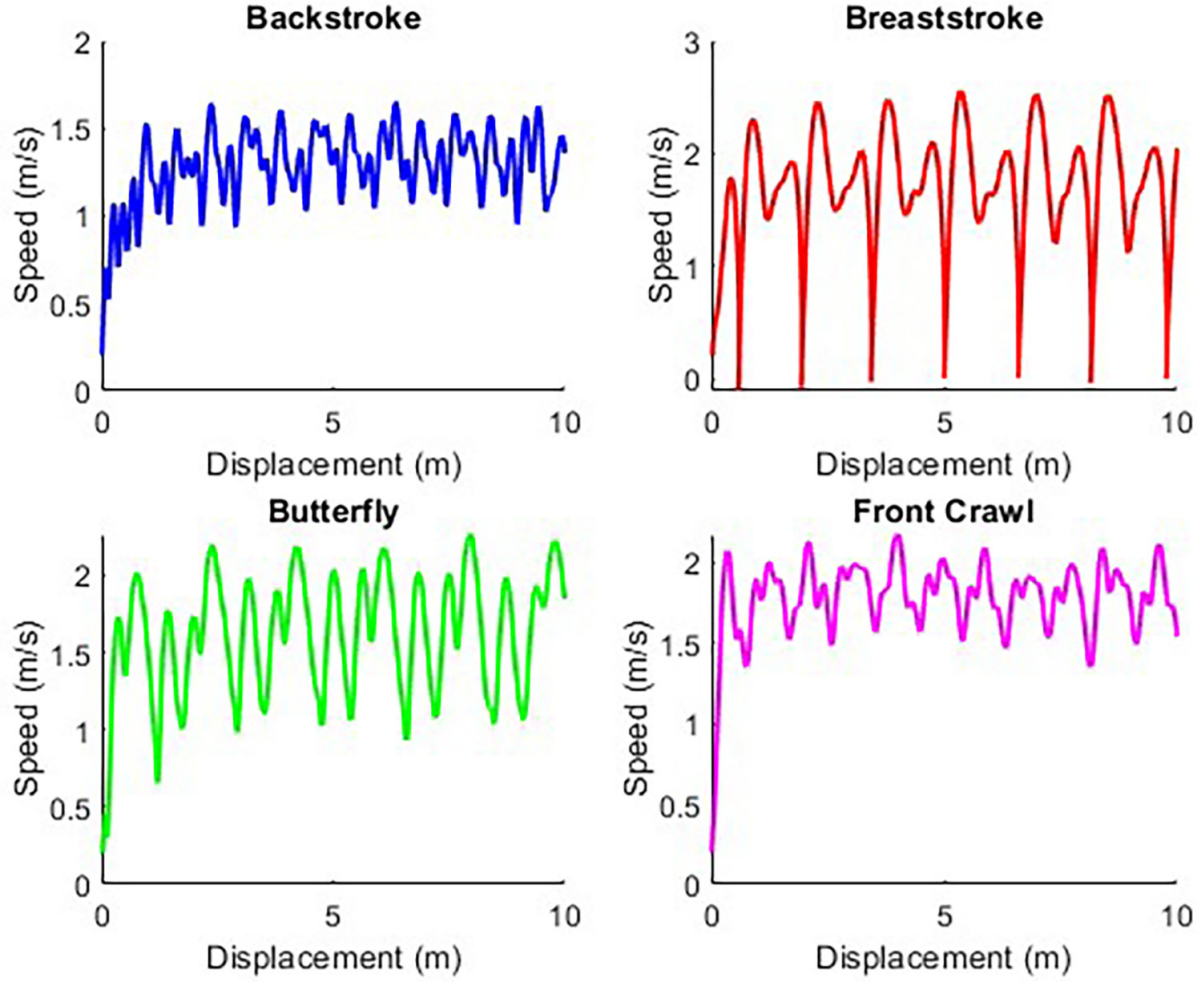
Examples of the speed output from the 1080 sprint in each swim stroke using a 1 kg resistance.

Position, force, and speed data, sampled at 333 Hz, were exported from 1080 Motion, and processed within a customized script (MATLAB R2023a, MathWorks, Natick, MA, USA). For all front-crawl and backstroke trials, the final 5 m of swimming was extracted for analysis. For breaststroke and butterfly trials, three consecutive stroke cycles within or near to the final 5 m of swimming were extracted (11, 18). Using stroke cycles instead of a fixed distance helps mitigate the risk of over- or underestimating swim velocity, as breaststroke and butterfly exhibit large velocity fluctuations that may not be fully captured over a set 5 m distance. These stroke cycles were identified using MATLAB's *findpeaks* function and manually selected based on stroke kinematics (34). Following horizontal correction, the average velocity was plotted against the corresponding load to create a load-velocity (LV) plot. Figure 2 presents a typical speed trace produced for each stroke with an external load of 1 kg. A linear regression line was established from each LV plot and extended to intercept the axes, identifying the theoretical maximal velocity (*V*_0_) theoretical maximal load (*L*_0_), with *L*_0_ also expressed relative to body mass (*rL*_0_). The relative slope between these variables calculated as −*V*_0_/*rL*_0_. Active drag (AD) was calculated using a modification of the velocity perturbation method (35) proposed by Gonjo and Olstad (20):

$$AD=\frac{F_{\mathrm{add}}\times V_{\mathrm{add}}\times V_{0}^{2}}{V_{0}^{3}-V_{\mathrm{add}}^{3}}$$

where *V*_0_ is derived from the LV relationship; *F*_add_ and *V*_add_ are the mean force and velocity extracted from the trial with the second heaviest load, which has been shown to exhibit higher reliability (20).

### Statistical analysis

Statistical analysis was performed using R (version 4.4.1) through Rstudio (version 2024.09.1). Data normality was assessed using the Shapiro–Wilk test. Linear mixed-effects models through *lme4* and *lmerTest* packages were used to assess the repeated measurements and account for missing observations (36, 37). The model was developed with maximum likelihood as the method of estimation and a random intercept. Dependent variables included Race Points, and LV outputs—*V*_0_, *L*_0_, the relative slope, and AD for both preferred-stroke and front-crawl. Fixed effects included observation, sex, and level (national or international), while swimmer was included as a random effect. Reference values were set as female swimmer, international level, at observation 1. The linear mixed-effects model is summarized as follows:

DependentVariable∼1+Observation+Sex+Level+(1|Swimmer)

Within-subject changes in preferred-stroke and front-crawl performance for *V*_0_ and *L*_0_ were evaluated by calculating the smallest worthwhile change (SWC) using the between-athlete standard deviation × 0.2 (38). The magnitude of change was assessed by plotting the point estimate with confidence intervals (CI) to determine whether the change was *positive, trivial,* or *negative* (39). If the CI spanned only one level, the change was considered *very likely positive, negative,* or *trivial.* If the CI spanned two levels, the change was classified as *possibly positive*, *negative*, or *trivial*, depending on the position of the point estimate. If the CI covered all three levels, the inference of change was deemed *unclear* (39). Pearson's correlations between LV outputs and race performance were assessed using the *hmisc* package. Correlation thresholds were interpreted as follows: 0.1 (small), 0.3 (moderate), 0.5 (large), 0.7 (very large), and 0.9 (extremely large) (37). Where possible, 95% CI are reported. Data visualizations were created using the *ggplot2* package. Statistical significance was defined as *p* ≤ 0.05.

## Results

**Figure 3:**
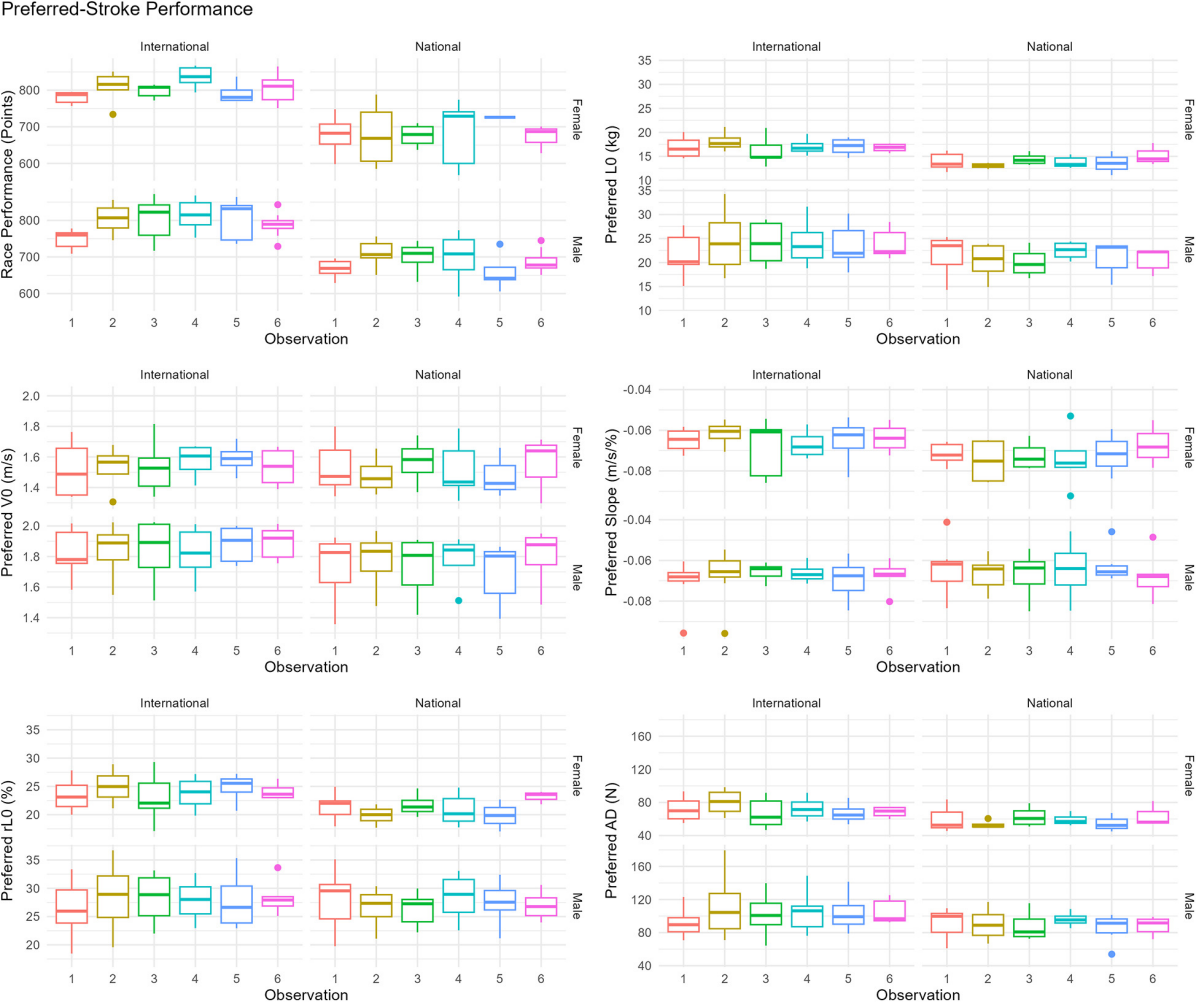
Box plots of preferred-stoke performance in competition and LV profiling.

**Figure 4:**
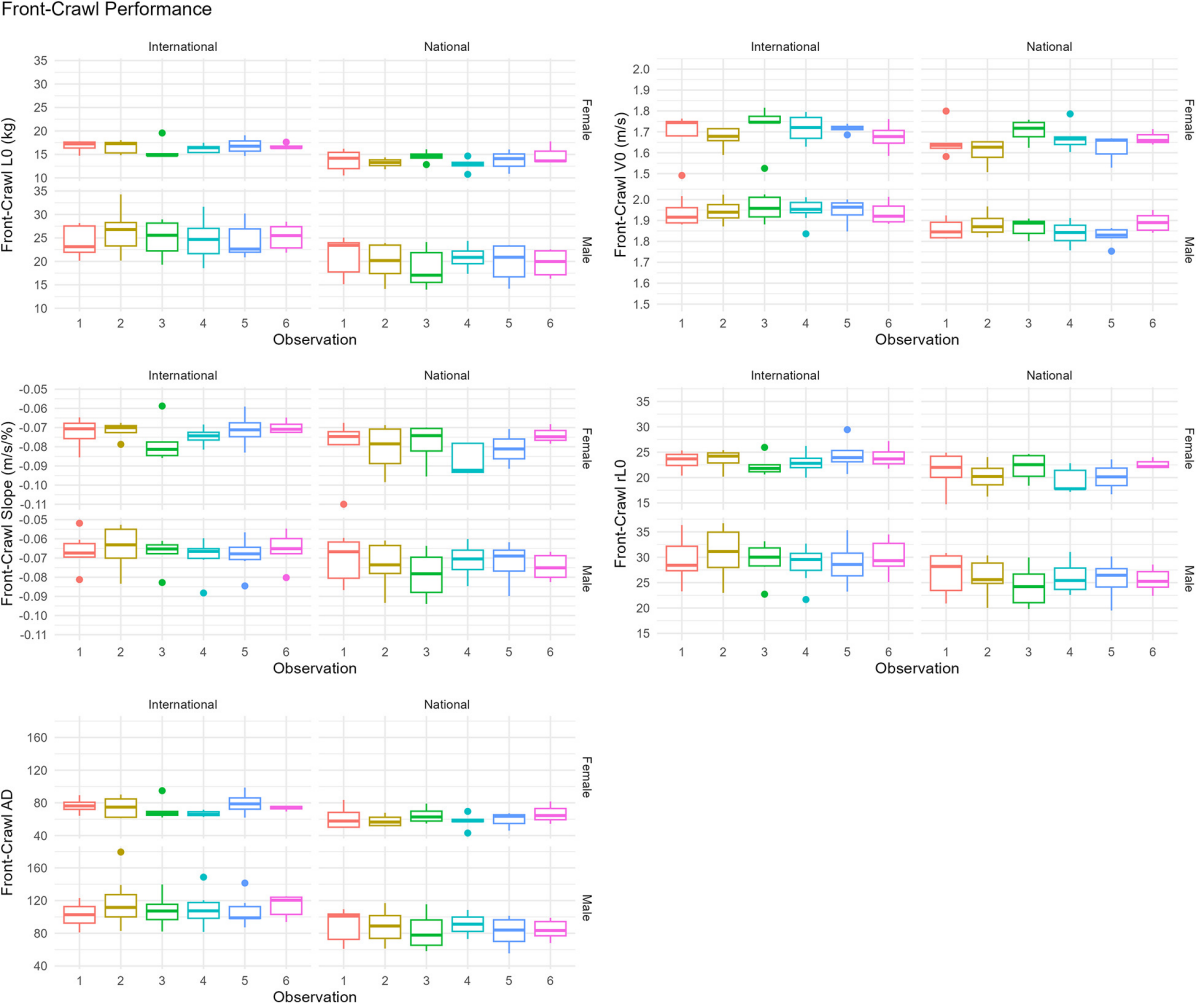
Box plots of front-crawl performance in LV profiling.

**Figure 5:**
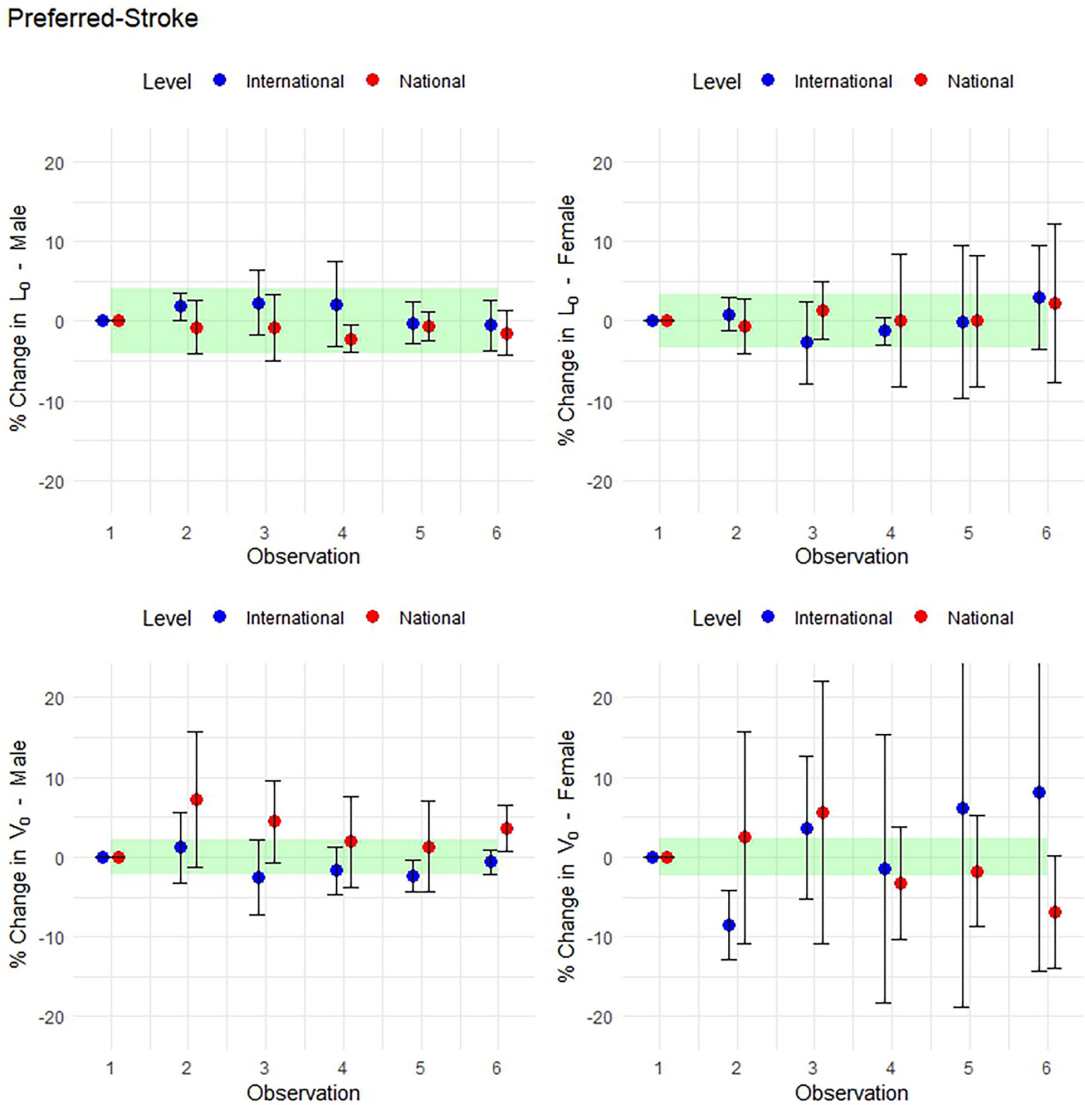
Mean within-athlete changes in preferred-stroke LV profiling. (Top row: *L*_0_ in males and females. Bottom row: *V*_0_ in males and females).

**Figure 6:**
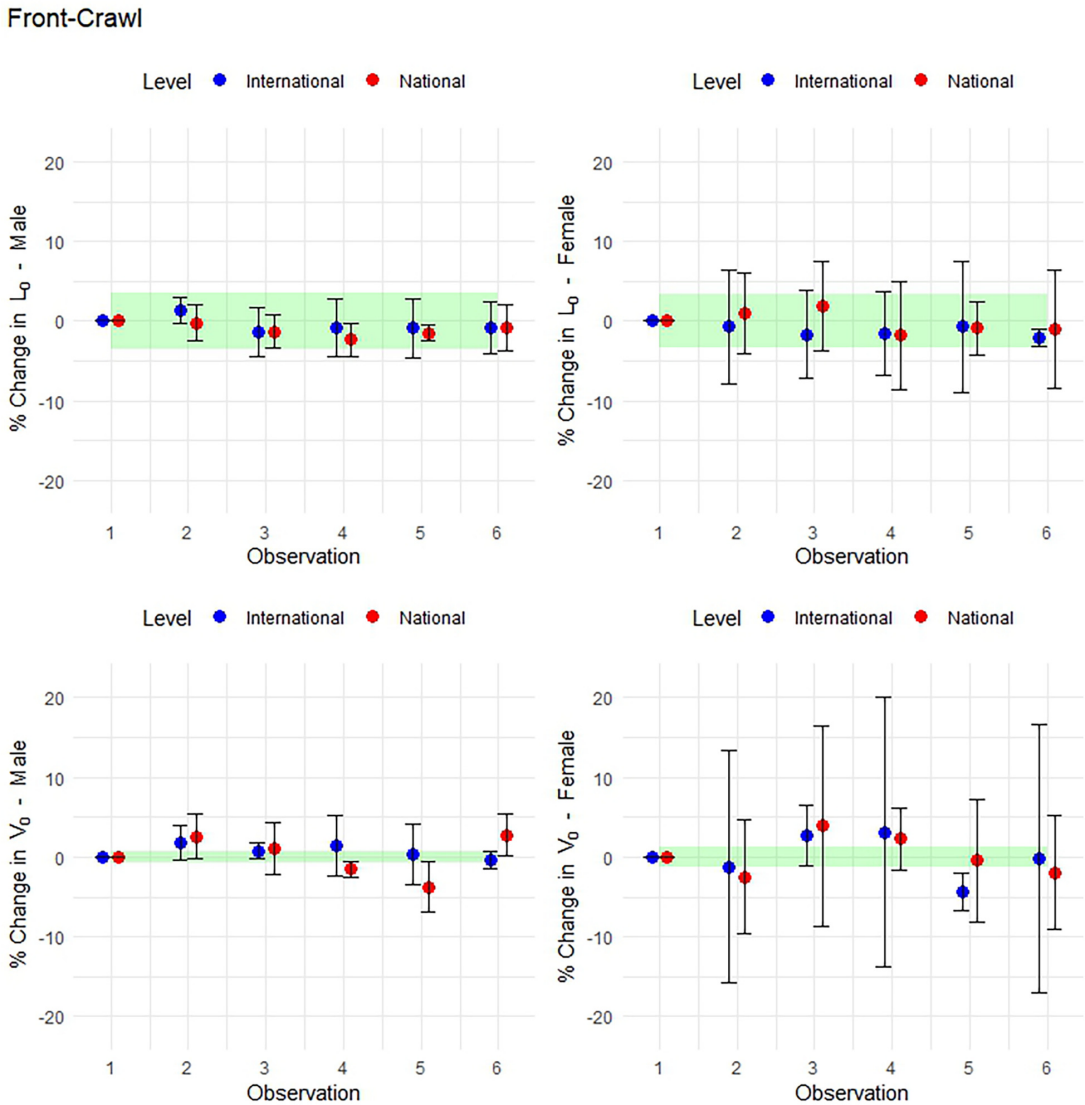
Mean within-athlete changes in front-crawl LV profiling. (Top row: *L*_0_ in males and females. Bottom row: *V*_0_ in males and females).

**Figure 7:**
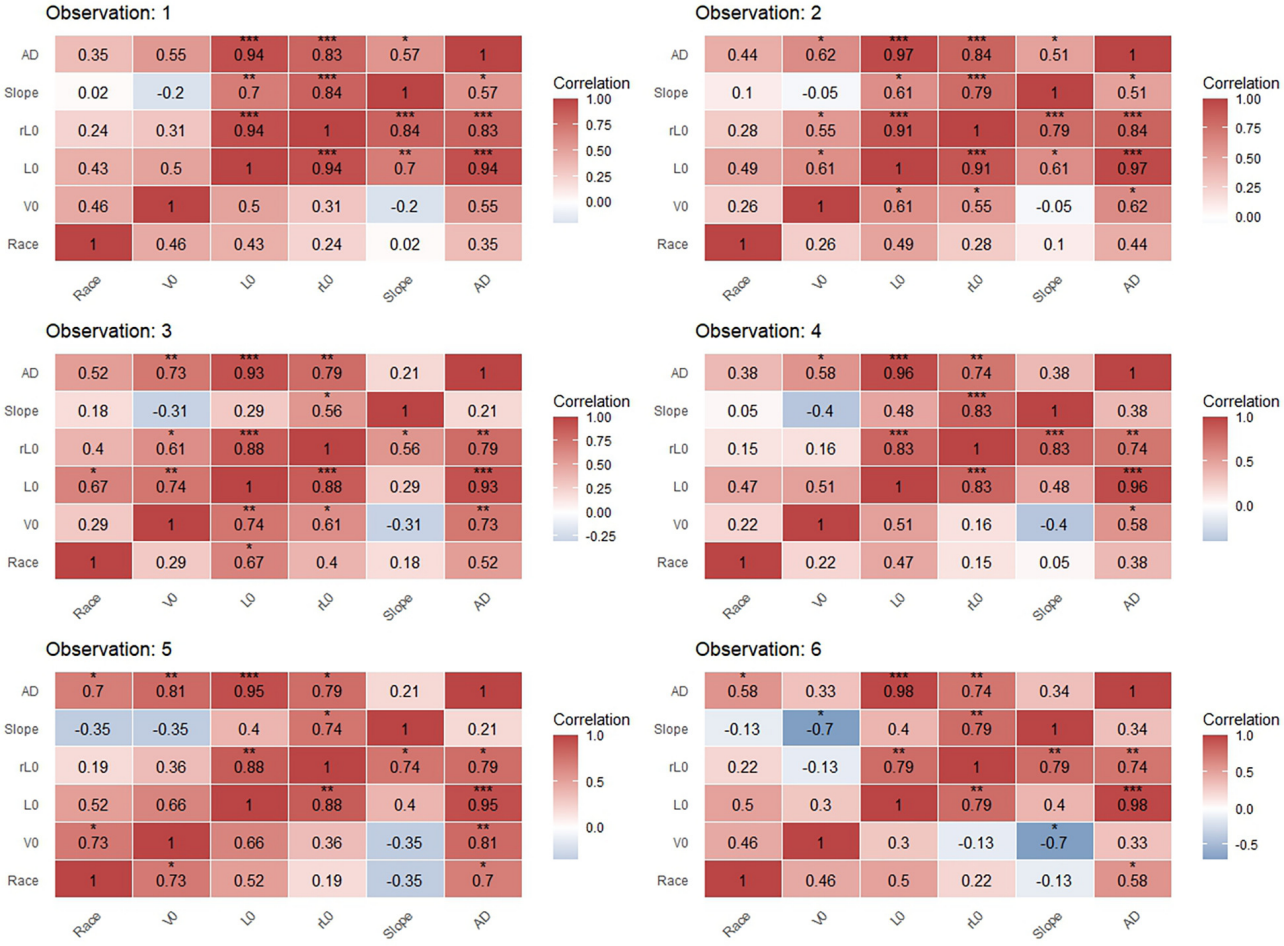
Correlation matrices between performance variables for males in preferred-stroke in competition and LV profiling (***p < 0.001, **p < 0.01, *p < 0.05).

**Figure 8:**
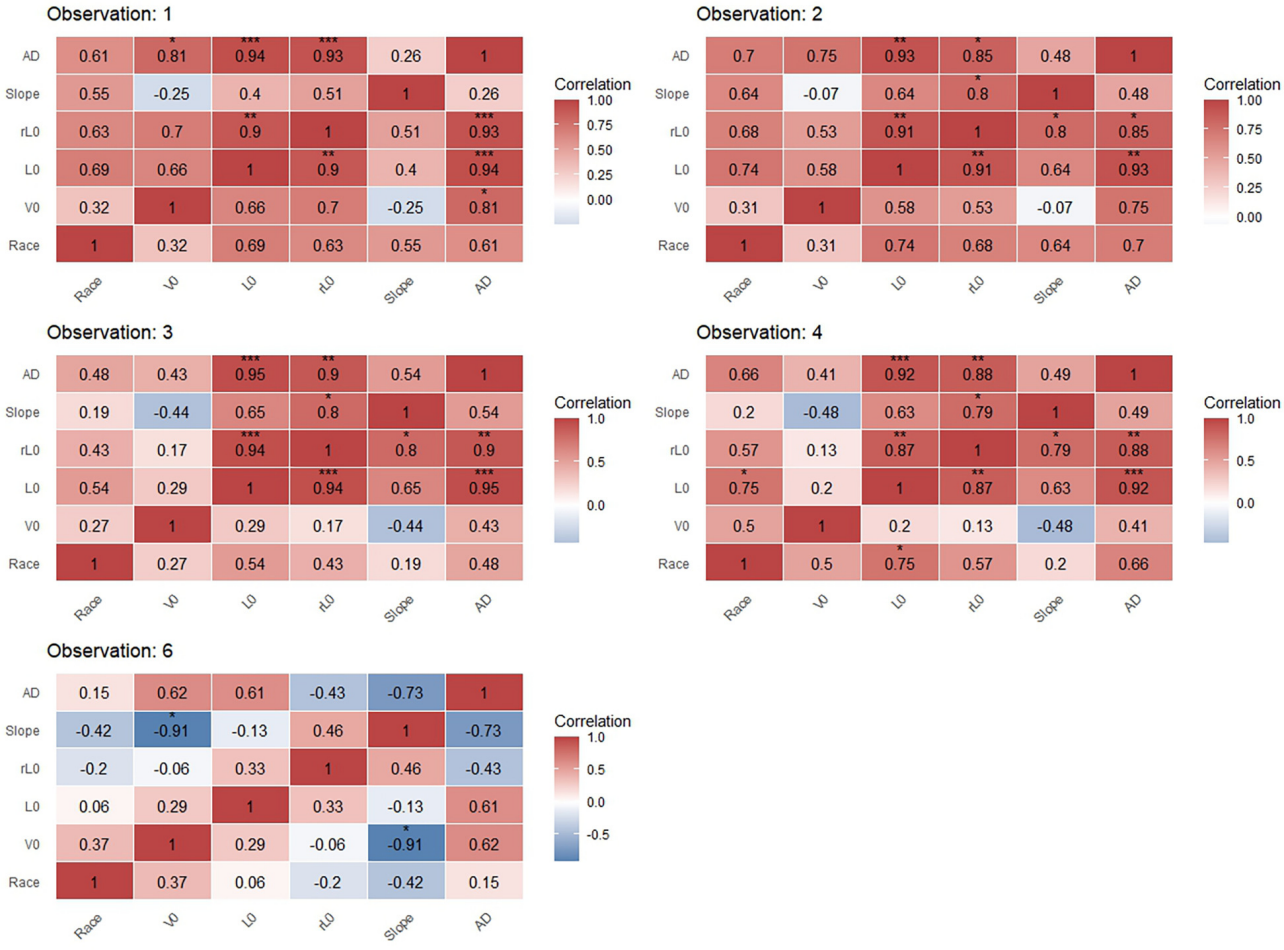
Correlation matrices between performance variables for females in preferred-stroke in competition and LV profiling (***p < 0.001, **p < 0.01, *p < 0.05).

Performance changes across all variables between males and female swimmers of national- and international-level, for both preferred-stroke and front-crawl, are presented as boxplots in Figures 3, 4, respectively. Average ± SD values for preferred-stroke were *V*_0_: 1.80 ± 0.17 and 1.54 ± 0.15 m/s; *L*_0_: 22.3 ± 4.2 and 15.5 ± 2.4 kg for males and females, respectively. For front-crawl performance, *V*_0_ was 1.91 ± 0.07 and 1.68 ± 0.08 m/s; *L*_0_: 22.6 ± 4.5 and 15.2 ± 2.1 kg, for males and females respectively. The results of the linear mixed-effects model, specifically the parameter estimates of fixed effects for both preferred-stroke and front-crawl, are presented in Tables 2, 3, respectively. Mean within-athlete changes in *V*_0_ and *L*_0_ for preferred-stroke and front-crawl performance are displayed in Figures 5, 6, respectively. Finally, correlation results between preferred-stroke performance variables for each observation are presented in Figures 7, 8 for males and females, respectively.

**Table 2:** Parameter estimates of fixed effects for race and load-velocity performance using preferred-stroke*.

Dependent Variable	Parameter		Estimate	SE	95% CI	df	*t*-value	*p*
Race performance (Points)	Intercept		769	15	739 to 798	31.7	50.8	<0.001
	Observation	Observation 2	40	8	25 to 55	113.9	5.3	<0.001
		Observation 3	37	8	22 to 52	113.9	4.8	<0.001
		Observation 4	45	8	30 to 60	113.7	5.9	<0.001
		Observation 5	14	9	−3 to 31	114.1	1.6	0.112
		Observation 6	25	8	10 to 40	114	3.3	0.002
	Sex	Male	2	16	−28 to 32	25.6	0.1	0.897
	Level	National	−113	15	−142 to −83	25.4	−7.5	<0.001
*V*_0_ (m/s)	Intercept		1.56	0.06	1.44 to 1.67	26.7	26.3	<0.001
	Observation	Observation 2	0.01	0.01	−0.01 to 0.03	102.0	0.9	0.362
		Observation 3	0.01	0.01	−0.02 to 0.03	102.1	0.5	0.623
		Observation 4	0.00	0.01	−0.03 to 0.02	102.1	−0.4	0.721
		Observation 5	0.00	0.01	−0.02 to 0.02	102.1	0.0	0.986
		Observation 6	0.01	0.01	−0.02 to 0.03	102.1	0.4	0.665
	Sex	Male	0.26	0.06	0.14 to 0.39	26.0	4.2	<0.001
	Level	National	−0.05	0.06	−0.18 to 0.07	26.0	−0.9	0.388
*L*_0_ (kg)	Intercept		16.8	1.1	14.6 to 19	31.2	14.9	<0.001
	Observation	Observation 2	0.5	0.5	−0.6 to 1.5	102.3	0.9	0.376
		Observation 3	−0.1	0.5	−1.1 to 1	102.4	−0.1	0.890
		Observation 4	0.2	0.5	−0.8 to 1.3	102.3	0.4	0.680
		Observation 5	−0.4	0.6	−1.5 to 0.8	102.9	−0.6	0.534
		Observation 6	−0.1	0.6	−1.2 to 1	102.9	−0.2	0.851
	Sex	Male	6.5	1.2	4.3 to 8.8	25.9	5.6	<0.001
	Level	National	−2.8	1.1	−5.1 to −0.6	25.9	−2.5	0.019
*rL*_0_ (%)	Intercept		23.4	1.2	21 to 25.8	32.1	19.1	<0.001
	Observation	Observation 2	0.3	0.6	−0.9 to 1.5	101.2	0.4	0.660
		Observation 3	−0.1	0.6	−1.3 to 1.2	101.4	−0.1	0.899
		Observation 4	−0.1	0.6	−1.4 to 1.1	101.3	−0.2	0.864
		Observation 5	−0.6	0.7	−2 to 0.7	102.1	−1.0	0.341
		Observation 6	−0.1	0.7	−1.4 to 1.2	102.0	−0.2	0.875
	Sex	Male	4.6	1.3	2.1 to 7.1	25.8	3.7	0.001
	Level	National	−1.5	1.2	−3.9 to 0.9	25.8	−1.2	0.244
Slope (m/s/%)	Intercept		−0.068	0.003	−0.074 to −0.061	33.5	−20.8	<0.001
	Observation	Observation 2	0.000	0.002	−0.003 to 0.004	101.5	0.2	0.870
		Observation 3	0.000	0.002	−0.004 to 0.004	101.7	0.0	0.977
		Observation 4	0.000	0.002	−0.004 to 0.004	101.6	0.0	0.969
		Observation 5	−0.002	0.002	−0.006 to 0.002	102.4	−0.9	0.361
		Observation 6	0.000	0.002	−0.004 to 0.004	102.3	0.1	0.934
	Sex	Male	0.002	0.003	0.005 to 0.004	25.9	0.5	0.607
	Level	National	−0.002	0.003	−0.008 to −0.004	25.9	−0.6	0.558
AD (N)	Intercept		70.5	6.1	58.6 to 82.4	31.8	11.6	<0.001
	Observation	Observation 2	5.0	3.0	−0.9 to 10.8	102.2	1.7	0.097
		Observation 3	0.2	3.1	−5.8 to 6.2	102.4	0.1	0.944
		Observation 4	1.5	3.1	−4.6 to 7.6	102.3	0.5	0.629
		Observation 5	−3.3	3.2	−9.6 to 3	102.9	−1.0	0.302
		Observation 6	−0.2	3.2	−6.5 to 6.1	102.9	−0.1	0.959
	Sex	Male	30.2	6.3	18 to 42.5	25.8	4.8	<0.001
	Level	National	−12.8	6.1	−24.7 to −0.9	25.7	−2.1	0.045

*V*_0_, theoretical maximum velocity; *L*_0_, theoretical maximum load; *rL*_0_, theoretical maximum load expressed as a percentage of body mass; AD, active drag, SE, standard error; CI, confidence intervals; df, degrees of freedom.

* The baseline (intercept value) was estimated for female swimmers, of international level at Observation 1.

**Table 3:** Parameter estimates of fixed effects for load-velocity performance using front-crawl*.

Dependent variable	Parameter		Estimate	SE	95% CI	df	*t*-value	*p*
*V*_0_ (m/s)	Intercept		1.70	0.02	1.66 to 1.74	32.5	82.2	<0.001
	Observation	Observation 2	0.00	0.01	−0.02 to 0.02	103.3	0.2	0.82
		Observation 3	0.02	0.01	0 to 0.04	103.6	1.8	0.076
		Observation 4	0.01	0.01	−0.01 to 0.03	103.4	1.0	0.325
		Observation 5	−0.01	0.01	−0.03 to 0.01	104.2	−0.8	0.45
		Observation 6	0.00	0.01	−0.02 to 0.02	104.2	0.1	0.907
	Sex	Male	0.23	0.02	0.18 to 0.27	25.8	10.6	<0.001
	Level	National	−0.06	0.02	−0.1 to −0.02	25.9	−3.1	0.004
*L*_0_ (kg)	Intercept		17.5	1.1	15.4 to 19.6	31.6	16.6	<0.001
	Observation	Observation 2	0.4	0.5	−0.6 to 1.4	103.2	0.7	0.463
		Observation 3	−0.7	0.5	−1.7 to 0.3	103.4	−1.4	0.179
		Observation 4	−0.7	0.5	−1.7 to 0.4	103.2	−1.3	0.204
		Observation 5	−0.6	0.6	−1.6 to 0.5	104.0	−1.0	0.318
		Observation 6	−0.5	0.6	−1.6 to 0.6	104.0	−0.9	0.372
	Sex	Male	7.2	1.1	5.1 to 9.3	25.7	6.6	<0.001
	Level	National	−4.3	1.1	−6.4 to −2.2	25.7	−4.1	<0.001
*rL*_0_ (%)	Intercept		24.3	1.1	22.1 to 26.5	33.2	21.9	<0.001
	Observation	Observation 2	0.2	0.6	−1 to 1.3	102.1	0.3	0.785
		Observation 3	−1.0	0.6	−2.2 to 0.3	102.5	−1.5	0.129
		Observation 4	−1.3	0.6	−2.5 to −0.1	102.1	−2.1	0.038
		Observation 5	−0.9	0.7	−2.2 to 0.4	103.2	−1.4	0.181
		Observation 6	−0.7	0.7	−1.9 to 0.6	103.1	−1.0	0.325
	Sex	Male	5.4	1.1	3.2 to 7.6	25.5	4.8	<0.001
	Level	National	−3.3	1.1	−5.5 to −1.2	25.5	−3.1	0.005
Slope (m/s/%)	Intercept		−0.072	0.003	−0.078 to −0.066	32.2	−21.6	<0.001
	Observation	Observation 2	0.001	0.002	−0.003 to 0.004	103.0	0.5	0.762
		Observation 3	−0.003	0.002	−0.007 to 0.001	103.3	−1.7	0.097
		Observation 4	−0.004	0.002	−0.008 to 0.000	103.0	−1.6	0.05
		Observation 5	−0.002	0.002	−0.006 to 0.002	103.9	−0.4	0.368
		Observation 6	−0.001	0.002	−0.005 to 0.003	103.9	−0.5	0.549
	Sex	Male	0.006	0.003	0.000 to 0.013	25.5	5.1	0.05
	Level	National	−0.008	0.003	−0.013 to −0.002	25.5	−4.0	<0.001
AD (N)	Intercept		77.5	5.5	66.8 to 88.3	34.4	14.1	<0.001
	Observation	Observation 2	3.9	3.2	−2.3 to 10.1	103.1	1.2	0.221
		Observation 3	−2.0	3.3	−8.4 to 4.5	103.6	−0.6	0.553
		Observation 4	−2.9	3.3	−9.3 to 3.5	103.2	−0.9	0.375
		Observation 5	−1.7	3.4	−8.4 to 5.1	104.3	−0.5	0.625
		Observation 6	−0.6	3.4	−7.4 to 6.1	104.3	−0.2	0.861
	Sex	Male	29.8	5.5	19.0 to 40.6	25.4	5.4	<0.001
	Level	National	−19.9	5.4	−30.4 to −9.3	25.5	−3.7	0.001

*V*_0_, theoretical maximum velocity; *L*_0_, theoretical maximum load; *rL*_0_, theoretical maximum load expressed as a percentage of body mass; AD, active drag, SE, standard error; CI, confidence intervals; df, degrees of freedom.

* The baseline (intercept value) was estimated for female swimmers, of international level at Observation 1.

### Preferred-stroke performance

As outlined in Table 2, using race performance at observation 1 as a baseline, there was a significant improvement in race points achieved in the following three competitions. However, no significant difference was observed at the competition aligned to observation 5, though performance improved again by observation 6. No significant difference in race points were found between male and female swimmers, but national-level swimmers achieved 14.7% fewer points than their international counterparts.

For the LV variables (*V*_0_, *L*_0_, *rL*_0_ and relative slope), no significant changes were observed across the six observations. However, males presented significant differences compared to females across all variables except the slope (*p* = 0.607). National-level swimmers demonstrated a 16.7% lower *L*_0_, but presented no statistical difference in *rL*_0_ (*p* = 0.244) or the slope (*p* = 0.558). While AD did not change significantly change across observations, males produced 42.8% greater drag, whereas national-level swimmers produced 18.2% less.

### Front-crawl performance

For front-crawl performance in LV profiling (Table 3), no significant differences in the variables were reported across observations, except for a reduction in *rL*_0_ and the slope at observation 4 (−1.3% and −0.004 m/s/%, respectively, *p* ≤ 0.05). Males presented a 13.5% faster *V*_0_, 41.1% larger *L*_0_, and 5.4% higher *rL*_0_, resulting in a 8.3% flatter slope. In contrast, national-level swimmers produced 3.5% lower *V*_0_, and 24.6% lower *L*_0_, leading to a 11.1% steeper slope. AD remained stable across observations, though males produced 38.5% greater drag, while national-level swimmers generated 25.7% less drag.

### Smallest worthwhile change in preferred-stroke performance

Using the SWC calculated from the between-athlete standard deviation at observation 1, the following mean within-athlete changes in *L*_0_ and *V*_0_ are shown across observations in Figures 5, 6 for preferred-stroke and front-crawl, respectively. Error bars represent 95% CI, indicating whether meaningful changes occurred relative to observation 1. In preferred-stroke LV performance, the magnitude of change of *L*_0_ for both international- and national-level males ranged from *very likely* to *possibly trivial* across all observations. For female, international-level swimmers, changes in *L*_0_ ranged from *very likely* to *possibly trivial* in observations 2, 3 and 4, and *unclear* for observations 5 and 6. For national-level females the level of change was *possibly trivial* at observations 2 and 3 and *unclear* for the remaining observations. For *V*_0_, national-level males are *possibly positive* at observations 2, 3 and 6 and *unclear* at observations 4 and 5. For international-level males, the level of change is *unclear* at observations 2, *possibly negative* at observations 3 and 5, and *possibly trivial* and *very likely trivial* at observations 4 and 6 respectively. For females, the level of change in *V*_0_ was *very likely negative* at observation 2 for international-level swimmers and *possibly negative* at observation 6 for national-level with *unclear* changes at all other observations.

### Smallest worthwhile change in front-crawl performance

In front-crawl LV performance (Figure 6), the magnitude of change of *L*_0_ for both international- and national-level males ranged from *very likely* to *possibly trivial* across all observations. Changes in *L*_0_ for females were *unclear* across all observations except being *possibly trivial* for national swimmers at observation 5 and *very likely trivial* for international swimmers at observation 6. For *V*_0_ in front-crawl, international-level males made *possibly positive* changes in observations 2 and 3 but *unclear* changes in the remaining observations. Change in national-level males was *possibly positive* at observation 2, *unclear* at observation 3, *very likely negative* at observation 4 and 5, then *possibly positive* at observation 6. For females, national-level changes across all observations were *unclear* while international-level were *unclear* at observations 2, 4 and 6, but *possibly positive* at observation 3 and *very likely negative* at observation 5.

### Correlation to race performance

Correlation results are presented in Figures 7, 8 for males and females, respectively. Results for females at observation 5 are excluded due to a small sample (*n* = 3) for both competition and profiling at that time-point. For male swimmers, significant, large to very large correlations existed between race performance and *L*_0_ at observation 3 (*r* = 0.67, *p* < 0.05); race performance and *V*_0_ and AD at observation 5 (*r* = 0.73 and *r* = 0.7, respectively, *p* < 0.05); and between race performance and AD at observation 6 (*r* = 0.58, *p* < 0.05). For female swimmers, a significant, very large correlation existed between race performance and *L*_0_ at observation 4 (*r* = 0.75, *p* < 0.05). No further statistically significant correlations were found between race performance and LV-derived variables in both sexes. Among LV-derived variables, *L*_0_, *rL*_0_ and *V*_0_ presented large to very large correlations (*r* = 0.55–0.74, *p* < 0.05) for males in observations 2 and 3. A very large and extremely large correlation was found between *V*_0_ and the slope in males (*r* = −0.7, *p* < 0.05) and females (*r* = −0.91, *p* < 0.05) at observation 6. Across both sexes and all observations except observation 6 in females, very large to extremely large correlations existed between AD and *L*_0_; and AD and *rL*_0_ (*r* = 0.74–0.97, *p* < 0.05).

## Discussion

This study monitored LV outputs (*V*_0_, *L*_0_, *rL*_0_, relative slope, AD), over a 15-month period in international- and national-level swimmers across both preferred-stroke and front-crawl swimming. While these variables fluctuated throughout the season, no statistically significant differences were found compared to baseline testing in preferred-stroke swimming. In front-crawl swimming, only a single observation showed a significant difference from baseline for *rL*_0_ and relative slope, respectively. Significant differences were evident between sexes and performance levels, with males and international swimmers tending to exhibit higher *L*_0_, suggesting superior force-generating capacity. These findings were supported by a magnitude-based assessment using the SWC to determine whether observed changes were practically meaningful. While individual analysis may provide further insight, group level changes assessed against the SWC suggest greater stability in international-level swimmers and more variability in national-level athletes, potentially reflecting differences in training consistency, recovery capacity, or adaptation to load. Despite limited correlations between LV outputs and race performance, large to very large correlations were found at specific time points between race performance and *V*_0_, *L*_0_, and AD, indicating that these variables may influence competitive outcomes over the course of a season. These findings suggest that while LV profiling alone is not a definitive predictor of race performance, it offers valuable insights into biomechanical adaptations and individual responses across time.

In LV profiling, *V*_0_ and *L*_0_ are theoretical extrapolations representing maximum velocity at zero load and maximum load at zero velocity, respectively. *V*_0_ serves as an indicator of free-swimming speed and *L*_0_ reflects the swimmer's propulsive force capabilities (11). While free-swimming speed in competition was not directly assessed, race performance (expressed as points) varied significantly over time, whereas *V*_0_ and *L*_0_ remained relatively stable. Changes in race performance ranged from 3 to 60 points across the six observations, equating to 0.02–0.43 s in the 50 m front-crawl, or 0.1–1.7 s in the 200 m front-crawl. Encouragingly, the progression of race points over the season aligns with the expectation of improved performances for international- and national-level swimmers towards key competition events (despite limited changes in LV outputs). It was also noted that the fastest performances occurred during the most important races in the Irish swimming calendar, indicating that training and tapering strategies were effective in preparing athletes for peak performance at the most critical moments. This underscores the multifaceted nature of race performance, which is influenced not only by free-swimming speed, but also by the start, turn(s), and finish phases (40). Given that the start and turn phases can account for nearly one-third of the total time in a 100 m event (40), improvements in these areas could enhance race performance independent of changes in free-swimming speed.

The LV slope, representing the relationship between *V*_0_ and *L*_0,_ has been suggested to be a strong indicator of AD (19), with steeper slopes associated with a greater ability to minimize resistance (17). In this study, the slope is expressed relative to body mass and calculated using *rL*_0_ to account for anthropometric and performance level differences. Interestingly, no significant difference in the LV slope was observed between sexes or performance levels in preferred-stroke swimming, despite males demonstrating higher *L*_0_, *rL*_0_, and faster *V*_0_. This suggests that while men produce greater absolute and relative loads, and reach higher velocities, the proportional relationship between load and velocity remains consistent across sexes. This supports previous findings that female swimmers may optimize force application through enhanced efficiency, focusing on minimizing resistance rather than generating higher propulsive force (41). Factors such as greater buoyancy, a more streamlined position, reduced frontal area, and technical adaptations to reduce drag may contribute to this efficiency. Similarly, the lack of slope difference between performance levels in preferred-stroke swimming, despite a higher absolute *L*_0_ in the international swimmers, suggests that this cohort applies force more effectively rather than simply increasing force production at the expense of efficiency. This highlights the importance of refining technique alongside strength development in high-performance swimming.

In front-crawl swimming, a key finding within this study cohort of 50–200 m specialists, was that while *V*_0_ did not differ between international and national-level swimmers, *L*_0_ and *rL*_0_ were significantly greater in the international-level group, resulting in a statistically flatter slope. This difference in force-generating capacity alters the shape of the LV profile, leading to a divergence in slope between performance levels. This suggests that while international swimmers generate greater propulsive force, they do so in a way that does not steepen the LV profile, potentially indicating more effective force application. This supports the idea that elite swimmers are not only stronger but also apply force more effectively to maintain velocity. This study proposes that an optimal LV profile in high-level sprint swimming may therefore be characterized by a high *V*_0_ (indicating greater swim speed) and high *L*_0_/*rL*_0_ (reflecting force-generation capacity), while maintaining a balanced slope that avoids unnecessary loss of effectiveness in force application. These findings highlight the importance of assessing not only the magnitude LV outputs but also the shape of the LV profile, which may serve as a valuable indicator of technical efficiency and training effectiveness across performance levels, offering coaches a more nuanced tool for tailoring strength and technique interventions in sprint swimming. Further research is required to determine whether this profile extends to swimmers specializing in longer distances.

Despite the lack of statistical differences in slope between sexes and performance levels in preferred-stroke analysis, differences in AD were evident across both preferred-stroke and front-crawl. Since AD is proportional to the square of velocity, an increase in swim velocity results in a quadratic increase in drag (42). Consequently, male swimmers and international-level athletes in this study, who achieved a statistically higher *V*_0_ than females and national-level athletes, are likely to experience larger drag forces. Similarly, a greater capacity for force generation, as indicated by a high *L*_0_ (11, 17), would lead to increased resistive forces in accordance with Newton's Third Law. However, rather than suggesting that females and national-level swimmers are inherently more efficient due to lower *L*_0_ values, these findings highlight the need to further explore *efficiency* in relation to both resistance reduction and propulsive force generation (43). Higher-performing swimmers may employ different strategies to optimize efficiency, balancing increased force output with effective drag management through biomechanical factors such as buoyancy, streamlined body positioning, and stroke mechanics (43, 44). Future research should examine how these elements contribute to overall efficiency across sex and different performance levels.

This study identified limited statistical changes in variables across observations, however, the application of magnitude-based inferences using the SWC provides additional insight into the degree of change within each observation. The SWC offers a useful benchmark for interpreting whether within-athlete performance changes exceed normal variation and reflect positive or negative trends (38). While group-based changes were generally small, especially among international-level swimmers, the presence of *possible* and *very likely* changes, particularly in *V*_0_, suggests meaningful trends that a *p*-value alone may overlook. Differences in change between males and females across different performance levels highlight the complex nature of swim performance. It is possible that meaningful changes were masked due to the range of strokes and distance specializations within each group. Interestingly, the group-based SWC analysis revealed greater variability among national-level swimmers, whereas international-level swimmers presented more stable performances, particularly in *L*_0_. This may reflect greater consistency in training, technical proficiency, or recovery strategies among higher level swimmers. Additionally, an *unclear* interpretation indicates that more data is required rather than the absence of change (39). Therefore, practitioners may benefit from assessing meaningful changes within more homogenous groups or by complementing group-level analyses with individual swimmer assessments to better capture nuanced performance trends. Furthermore, comparing profiling results alongside training data, specifically any details of in-water resistance training, may provide further insight into the extent and causes of performance changes.

Although training data was not collected as part of this study, a two-year observation of the training centers where these athletes train revealed that swimmers typically complete 6–10 pool sessions per week, along with an average of two strength and conditioning sessions per week (45). While strength was not directly assessed, international-level swimmers in this study were statistically older and heavier, suggesting a longer competitive training history, greater accumulated training volume, and increased exposure to high-level coaching. These factors likely contribute to skill execution in the start and turn phases, while increased lean muscle mass may enhance strength and power, both of which are correlated with improved start and turn performance (46, 47). Furthermore, land-based strength and conditioning routines have been shown to positively impact stroke length, while in-water resistance training may improve stroke rate (48), with both variables associated with greater swim performance (49, 50). This study did not measure race components in terms of stroke length and stroke rate. Future studies should incorporate these metrics to gain a more comprehensive evaluation of seasonal performance variations.

Despite the lack of consistent relationships between LV outputs and race performance (as measured by race points), several cross-sectional studies in sprint swimming support the relevance of LV profiling. In a 50 m short-course time-trial involving Level 3 and 4 swimmers (31), *V*_0_ and *L*_0_ were significantly correlated to finishing time in front-crawl (*r* = −0.68 and −0.55, respectively), backstroke (*r* = −0.71 and −0.72), butterfly (*r* = −0.81 and −0.62), and breaststroke (*r* = −0.52 and −0.62,) (11, 16–18). Similarly, in Level 1–3 swimmers performing their preferred stroke, females exhibited a strong correlation between *V*_0_ and a 50 m time-trial (*r* = −0.96), while *L*_0_ showed a weaker, non-significant correlation (*r* = −0.64) (Keating et al.). For males, both *V*_0_ and *L*_0_ were significantly correlated with a 50 m time-trial (*r* = −0.71 and −0.62, respectively) (Keating et al.). However, findings were less consistent in a more homogenous sample of Level 1–2 swimmers, with limited significant correlations observed (13). Specifically, *rL*_0_ and the absolute slope showed very largely correlations with stroke rate in 100 m events, while *V*_0_ was reported to have a very large negative correlation with stroke length in 200 m races (13). These findings suggest LV profiling may be more relevant for sprint specialists, though its applicability to distance swimming remains unexplored. It is plausible that distance swimmers present distinct LV profiles characterized by lower values of *V*_0_ and *L*_0_ but with a greater capacity of sustaining these outputs over time.

While LV performance alone does not determine race performance, its relevance to sprint swimming warrants further exploration. LV profiling is a time-efficient method for tracking key variables of *V*_0_, *L*_0_, and slope over time. However, optimizing these variables through training remains an area for future research. Evidence from sprint running underscores the benefits of LV profiling in load prescription interventions. Specifically, individualized LV profiles have been used to prescribe training loads corresponding to velocity decrements of 25, 50, and 75%, targeting speed-strength, power, and strength-speed adaptations, respectively, in sled pushing and pulling (51, 52). Additionally, Petrakos et al. (53) proposed categorizing resistance training loads based on velocity decrements of <10, 10–15, 15–30, and >30% as light, moderate, heavy, and very heavy, respectively. While exact threshold values may vary depending on the targeted adaptation, velocity-decrement-based prescription is gaining traction (54) and may hold potential for enhancing adaptations in swimming-specific training programs. It is important to note that the present study was not designed to assess a prescribed intervention targeting LV outputs. Instead, it was aimed to examine how these variables naturally evolve within independent training plans across multiple high-performance sites, each with distinct coaching philosophies. Future research should focus on developing a targeted in-water resistance training intervention and evaluating its impact on LV outputs and race performance. By doing so, it may be possible to refine training methodologies that optimize LV characteristics for improved competitive outcomes in sprint swimming. Furthermore, integrating measures such as stroke kinematics, coordination patterns, and blood lactate responses into the LV profiling protocol may provide a more comprehensive understanding of swimmer adaptations and training effects.

This is the first study to assess LV performance in national- and international-level swimmers over a 15-month period. The application of linear mixed-effect models in the statistical analysis represents best practice in sports science (36, 37), allowing for the inclusion of incomplete repeated measures. However, several limitations within the study must be acknowledged: (i) Due to the decentralized, real-world training structure, where swimmers trained at different locations under various coaches, meant that detailed training data were not recorded, limiting our ability to assess its influence on LV outputs. However, this approach provided a valuable opportunity to observe high-level athlete adaptations during critical phases of the competitive season, including preparation for Olympic selection trials; (ii) Race points were used as a standardized measure of performance across long- and short-course competitions over a 15-month period. While commonly employed within the coaching community, conversion calculators for swim times lack academic validation. Additionally, race points do not account for changes in start, turn, or kinematic variables such as stroke length and stroke rate; (iii) Combining different strokes and distances may have masked stroke- or distance-specific changes in LV outputs. Future research should investigate whether targeted interventions aimed at improving *V*_0_ and/or *L*_0_ lead to meaningful changes in LV outputs and, ultimately, race performance. Additionally, monitoring stroke rate and stroke length will be critical for identifying potential adaptations contributing to performance improvements.

## Conclusion

This study monitored changes in LV outputs in both preferred-stroke and front-crawl swimming among national- and international-level swimmers over a 15-month competitive season. While LV outputs fluctuated over time, statistically significant changes compared to baseline were limited. Notable differences emerged between sexes and performance levels, with males and international-level swimmers demonstrating greater *L*_0_, suggesting superior force-generating capacity. However, similar *rL*_0_ values between performance levels indicate this was achieved without compromising efficiency.

Although LV profiling alone is not a direct predictor of race performance, our findings reinforce its value as a tool for monitoring biomechanical adaptations and individual responses over time. These metrics may inform individualized training strategies by identifying meaningful trends that traditional statistics may overlook. Future research should explore targeted interventions designed to enhance *V*_0_, *L*_0_, and the slope, and assess their impact on technical and physiological adaptations.

Additionally, incorporating AD monitoring and stroke kinematic measures, such as coordination patterns and hand velocity, may provide a more comprehensive understanding of performance development in sprint swimming. These findings highlight the potential of LV profiling to contribute to training prescription, talent identification, and long-term athlete development in high-performance environments.

## Data Availability

The raw data supporting the conclusions of this article will be made available by the authors, without undue reservation.
